# Quality of life and physical activity in long-term (≥5 years post-diagnosis) colorectal cancer survivors - systematic review

**DOI:** 10.1186/s12955-018-0934-7

**Published:** 2018-06-01

**Authors:** Ruth Elisa Eyl, Kun Xie, Lena Koch-Gallenkamp, Hermann Brenner, Volker Arndt

**Affiliations:** 1 German Cancer Research Center (DKFZ), Division of Clinical Epidemiology and Aging Research, Im Neuenheimer Feld 581, 69120 Heidelberg, Germany; 2 University of Heidelberg, Medical Faculty Heidelberg, Im Neuenheimer Feld 672, 69120 Heidelberg, Germany; 3 German Cancer Research Center (DKFZ) and National Center for Tumor Diseases (NCT), Division of Preventive Oncology, Im Neuenheimer Feld 460, 69120 Heidelberg, Germany; 4 German Cancer Research Center (DKFZ), German Cancer Consortium (DKTK), Im Neuenheimer Feld 280, 69120 Heidelberg, Germany; 5 German Cancer Research Center (DKFZ), Divison of Clinical Epidemiology and Aging Research, Unit of Cancer Survivorship, Im Neuenheimer Feld 581, 69120 Heidelberg, Germany

**Keywords:** Colorectal cancer, Quality of life, Physical activity, Exercise, Cancer survivor

## Abstract

**Background:**

Due to the increasing number of long-term (≥5 years post diagnosis) colorectal cancer survivors, long-term quality of life of these patients is highly relevant. Several studies have reported a positive association between physical activity and quality of life in colorectal cancer survivors, however, so far no systematic review has been published which focuses on long-term colorectal cancer survivors.

**Material and methods:**

A systematic review was conducted using the databases PubMed, Web of Science, PsychINFO, and CINAHL. Studies which investigated associations between physical activity and quality of life in long-term colorectal cancer survivors were included.

**Results and conclusion:**

Ten articles based on seven studies were identified. Long-term colorectal cancer survivors who were physically active reported better quality of life than long-term survivors who were not physically active. Both, moderate to vigorous physical activity and lower levels like light physical activity were associated with higher quality of life. Most studies assessed the association between physical activity and quality of life cross-sectionally but one prospective study which measured physical activity and quality of life at three different points in time also found associations between physical activity and quality of life. The association between physical activity and quality of life seemed to be stronger among women than among men. The findings of this systematic review support an association between physical activity and quality of life in long-term colorectal cancer survivors. However, the evidence is limited as most studies were based on cross-sectional and observational design.

**Electronic supplementary material:**

The online version of this article (10.1186/s12955-018-0934-7) contains supplementary material, which is available to authorized users.

## Background

In 2012, there were almost 1.4 million incident cases and roughly 700,000 deaths due to colorectal cancer (CRC) worldwide [1]. Colorectal cancer is the second most common cancer and the second leading cause of cancer-related deaths in Europe [2].

There is strong evidence that physical activity (PA), in particular leisure-time PA, is associated with better overall [3–6] as well as CRC-specific [4, 5] survival in CRC patients. According to a recent meta-analysis based on 7422 CRC patients, PA after diagnosis was associated with a 39% lower risk of CRC-specific mortality [5]. Moreover, evidence from several studies [7–9] further suggests that PA might have a positive effect on quality of life (QOL) in CRC survivors. Studies have shown that patients who were more physically active tended to report better QOL, better functioning, less pain, insomnia, and fatigue [8, 9]. However, a recent review article by Lynch et al. [10] reported inconsistent results of studies which investigated the association between PA and QOL in short-term and long-term (≥5 years post-diagnosis) CRC survivors. Although observational studies unanimously observed associations between PA and QOL, the evidence is much weaker from intervention studies. No systematic review to date has focused specifically on associations between PA and QOL in long-term CRC survivors.

Due to recent improvements in early detection and treatment, the 5 year survival-rate of CRC has increased up to 66% [11, 12]. Thus, the QOL of long-term CRC survivors is a highly relevant issue. Although a number of studies [13–15] found that the overall QOL of CRC survivors was comparable to the general population, they also reported that CRC survivors experience detriments in symptom-related QOL, even years after diagnosis. Studies [13, 16] further suggest that detriments in QOL might be largest among younger CRC survivors compared to cancer controls. Also, the QOL of CRC survivors has been shown to change throughout the years after diagnosis. Jansen et al. found that facets of QOL, such as physical functioning and pain worsened over a 10-year follow-up period [16]. Moreover, it has been reported that CRC survivors experience different psychosocial and physical symptoms at various points in time after diagnosis; for example shortly after treatment survivors reported more frequently symptoms such as neuropathy and sleep difficulty [17] whereas long-term CRC survivors reported to have symptoms such as bowel problems, stress related to cancer, and depression [18]. Due to these differences in psychosocial and physical symptoms between short-term and long-term survivors, we hypothesize that the overall effect of PA on QOL might vary.

As PA may represent a promising intervention to improve QOL and alleviate the burden of living with cancer and since there has not been much research in this field, this review summarizes the current available evidence investigating the association between PA and QOL in long-term CRC survivors.

## Materials and methods

The literature search was carried out in August 2016 and was repeated in January 2017 to guarantee inclusion of all relevant publications. The databases PubMed, Web of Science, PsychINFO and CINAHL were searched for relevant articles. The exact combinations of search terms are listed in the Additional file 1: Table S1. Cross-referencing was performed to identify additional articles which were not identified by the database search.

### Inclusion criteria

To be included in the review, studies had to assess QOL in CRC patients 5 and more years post-diagnosis and PA within the time span of diagnosis to QOL assessment. Results of studies which investigated short-term as well as long-term survivors were also eligible if specific results for long-term survivors were provided. Studies comprising survivors with a mean of ≥5 years since diagnosis were also included. We did not include studies examining PA/QOL among CRC survivors regardless of time since diagnosis, since testing for a moderating effect of time was not our major interest. All types of CRC and all kinds of PA were eligible. However, QOL had to be assessed by more than one scale as it is a multidimensional concept. When studies investigated several cancer types, only the specific results for CRC survivors were included. Furthermore, PA had to be the independent variable and QOL the outcome. All types of quantitative original studies, published in English or German, were included. Conference abstracts, study protocols, editorials, commentaries, qualitative studies, theses, reviews, and meta-analyses were not considered. There was no restriction regarding the publication date.

### Data extraction

Titles and abstracts of all identified articles were screened by the first reviewer (RE). Subsequently the full texts of the selected articles were checked for eligibility. The study characteristics of the eligible studies (e.g. first author, year, journal, sample size, country, sex, age, tumor site, cancer stage, cancer treatment, sampling, study design, comorbidities, inclusion and exclusion criteria, baseline response rate, timing/type of PA assessment, timing/type of QOL assessment, confounders/adjustment, statistical methods, results) were independently extracted by two reviewers (RE and KX). Discrepancies were discussed and if they could not be solved, a third reviewer (VA) was involved.

### Statistical significance and clinical relevance

All statistically significant results mentioned in this review refer to a *p*-value <0.05. If studies reported clinical relevance using either the European Organization for Research and Treatment of Cancer QLQ-C30 questionnaire (EORTC QLQ-C30) or the Short Form Health Survey (SF-36), the reported clinical relevance was adopted. For those studies using the EORTC QLQ-C30 and not reporting clinical relevance, we determined clinical relevance by using a medium clinical relevance, which is defined by Osoba et al. [19] as a mean difference of ≥10 score points.

### Combining the results of different QOL instruments

As the included studies used various QOL instruments with different notation for the embedded scales, results pertaining different QOL scales of different questionnaires were combined as shown in Table 1.

**Table 1:** Combining the results of different QOL instruments

	Questionnaire	Scale
Global QOL	EORTC QLQ-C30 [45]	Overall QOL/ global health
	SF-36 [46]	General health and global health composite score
	EQ-5D [47]	Overall health related quality of life (HRQOL)
Physical functioning	EORTC QLQ-C30	Physical functioning
	SF-36	Physical functioning and physical health composite score
	FACT-C [48]	Physical well-being
	PROMIS [49]	Physical HRQOL
Role functioning	EORTC QLQ-C30	Role functioning
	SF-36	Role physical
	FACT-C	Functional well-being
Social functioning	EORTC QLQ-C30	Social functioning
	SF-36	Social functioning
	FACT-C	Social well-being
Emotional functioning	EORTC QLQ-C30	Emotional functioning
	SF-36	Mental health
	FACT-C	Emotional well-being

Two reviewers (RE and KX) checked the methodological quality of each included article using items adapted from the checklist of Mols et al. [20], with a more detailed emphasis on contents that are important to the specific study question of this review (Table 2). The following quality criteria were considered:

1. Information bias:
  - Adequate assessment of exposure (i.e. valid PA instrument, assessment of all PA aspects, objective measure rather than self-report)
  - Adequate assessment of outcome (i.e. valid QOL instrument, assessment of all relevant QOL aspects)
  - Adequate description of data (socio-demographic and medical data is described e.g. age, tumor stage at diagnosis etc.; the process of data collection is described e.g. interview or self-report)
2. Selection bias:
  - Inclusion and/or exclusion criteria are formulated
  - Healthy (survivor) participation bias (i.e. information about non-participants at baseline, information about drop-outs at follow-up, attrition bias)
3. Study design:
  - Description of timing of PA/QOL assessment
  - Adequate information regarding time since diagnosis
  - Adequate sample size and power
  - Prospective study design rather than cross-sectional
4. Correction of outcome measures for confounding (e.g. age, sex, comorbidities)

**Table 2:** Quality assessment of included studies

First author year (ref.) country	Potential Limitations
Blanchard 2004 [35] USA	- No validated PA questionnaire used
	- Possible response bias due to self-reported PA
	- Sample size < 100
	- Cross-sectional study design
Blanchard 2008 [30]^a^ USA	- Possible response bias due to self-reported PA
	- Only assessment of leisure-time PA
	- Cross-sectional study design
Blanchard 2010 [31]^a^ USA	- Possible response bias due to self-reported PA
	- Only assessment of leisure-time PA
	- Cross-sectional study design
Chambers 2012 [38] Australia	- Possible response bias due to self-reported PA
	- Only assessment of leisure-time PA
Husson 2015 [34]^b^ The Netherlands	- Possible response bias due to self-reported PA
Mols 2015 [9]^b^ The Netherlands	- Possible response bias due to self-reported PA
	- Cross-sectional study design
Rodriguez 2015 [36] USA	- Possible response bias due to self-reported PA
	- Cross-sectional study design
Thraen-Borowski 2013 [37] USA	- Possible response bias due to self-reported PA
	- Only assessment of leisure-time PA
	- Cross-sectional study design
Van Roekel 2015 [32]^c^ The Netherlands	- Possible response bias due to self-reported PA
	- Cross-sectional study design
Van Roekel 2016 [33]^c^ The Netherlands	- Cross-sectional study design

^a^Articles based on same study population: American Cancer Society’s Study of Cancer Survivors-II (SCS-II); ^b^Articles based on same study population: All patients diagnosed between 2000 and 2009 and registered in the Patient Reported Outcomes Following Initial treatment and Long term Evaluation of Survivorship (PROFILES registry); ^c^Articles based on same study population: Energy for life after ColoRectal cancer (EnCoRe)

This systematic review was guided by the criteria, set out by the PRISMA guidelines [21].

## Results

### Literature search

**Fig. 1:**
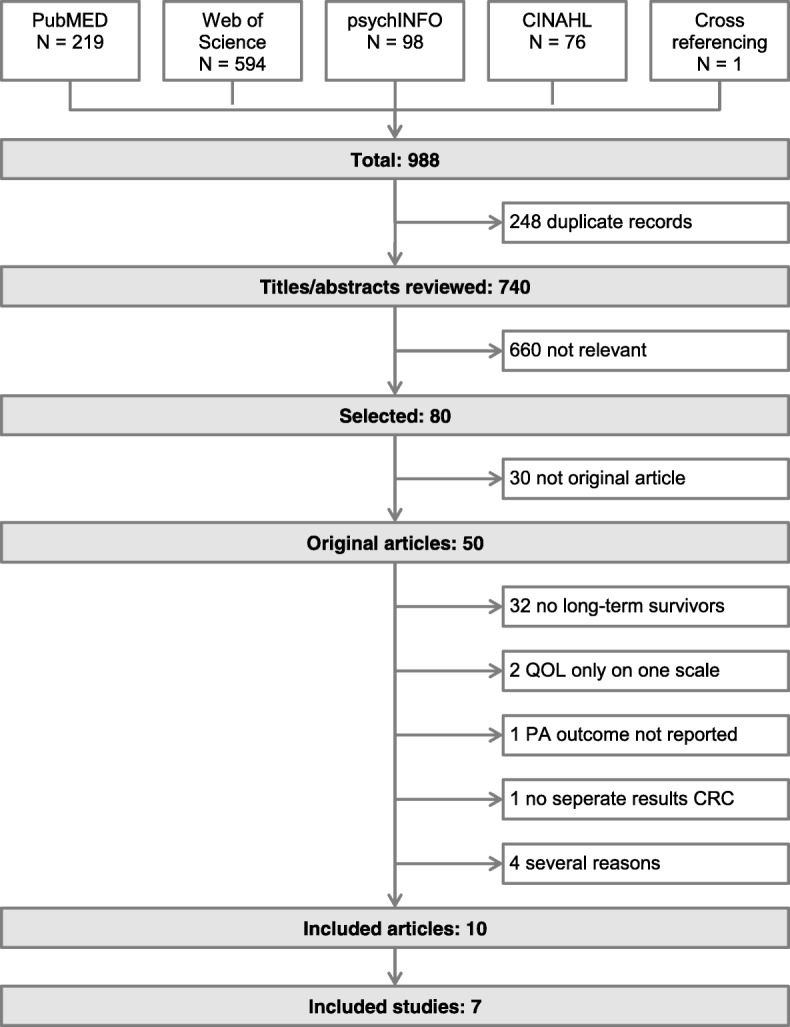
Literature search process. QOL: quality of life; PA: physical activity; CRC: colorectal cancer

The search identified 988 articles (Fig. 1). After removing the duplicates, 740 publications remained. After checking titles and abstracts for eligibility, 80 relevant articles were identified. Thirty articles were excluded because they were not original articles, and 32 were excluded because they did not include long-term CRC survivors. Two studies [22, 23] assessed QOL on only one scale and were therefore excluded. One study [24] did not report any results regarding the association of PA and QOL. One study [25] did not report separate results for CRC survivors and four studies [26–29] were excluded for several other reasons. In the end, ten articles based on seven studies were included in this systematic review. Two articles of Blanchard et al. [30, 31] were based on the same study population (American Cancer Society’s Study of Cancer Survivors-II, SCS-II). Also the data for the two articles of van Roekel et al. [32, 33] were taken from an identical study population (Energy for life after ColoRectal cancer, EnCoRe). Further, all CRC patients diagnosed between 2000 and 2009 as registered in the PROFILES cancer registry were selected for the articles of Mols et al. [9] and Husson et al. [34]. In case of multiple articles per study, each study only counted once but results from all articles are shown in the tables.

### Study characteristics

#### Participants’ characteristics

Four studies were conducted in the US [30, 31, 35–37], two [9, 32–34] in the Netherlands and one in Australia [38] (Table 3). Sample sizes ranged from 86 [35] to 1918 [30]. All of the included studies investigated female and male survivors, but most reported a slightly higher proportion of males. The mean age at time of QOL assessment ranged from 68.4 [34] to 81.5 [37] years. Two studies were restricted to long-term survivors only [36, 37]. All the other studies [9, 30–35, 38] did not provide specific results for long-term CRC survivors, but comprised survivors with a mean of ≥5 years since diagnosis at the time of QOL assessment. Four studies [30–35] included CRC survivors from 2 years post-diagnosis, one prospective study [38] included participants from five months post-diagnosis, but the results for the association between PA and QOL was based on PA and QOL data collected 5 years post-diagnosis. Mols et al. [9] included survivors from 1 year up to 11 years post-diagnosis.

**Table 3:** Study characteristics

First author year (ref.) country	Study design	Sample size	Age at survey	Time since diagnosis^a^	Cancer treatment	Cancer stage	PA instrument	QOL instrument	Meeting ACS PA guideline
Blanchard 2004 [35] USA	Cross-sectional, population-based	86	Mean(SD) 69.22(12.5)	≥2 years 33.7% ≥5 years 30.2% ≥10 years 36.0%	Surgery Radiation Chemotherapy	I-IV	Adherence to ACS PA guideline	SF-36	69.8%
Blanchard 2008 [30]^b^ USA	Cross-sectional, population-based	1918	Mean(SD) 70.2(11.0)	≥2 years 33.4% ≥5 years 35.3% ≥10 years 31.3%	Surgery Radiation Chemotherapy Hormone therapy Immuno therapy BMT	I-IV	GLTEQ	SF-36	35%
Blanchard 2010 [31]^b^ USA	Cross-sectional, population-based	668	Mean(SD) 70.2(11.1)	≥2 years 26.8% ≥5 years 40.5% ≥10 years 32.0%	In treatment (not further specified)	I-IV	GLTEQ	SF-36	HW 20.0% OW 30.0% OB 24.4%
Chambers 2012 [38] Australia	Cross-sectional & longitudinal, population-based	632	Mean 69.02	≥5 years Mean(SD) 5(6.1)	Surgery Chemotherapy	I-III	AAS	FACT-C SWLS	–
Husson 2015 [34]^c^ The Netherlands	Cross-sectional & longitudinal, population-based	1739	Mean(SD) 68.4(9.4)	≥2 years Mean(SD) 5.1(2.8)	Radiation Chemotherapy	I-IV	EPIC	EORTC QLQ-C30	82%
Mols 2015 [9]^c^ The Netherlands	Cross-sectional, population-based	1648	Mean(SD) Chemotherapy: 66.7(9.8) No chemotherapy: 70.6(9.0)	1–11 years Mean(SD) Chemotherapy: 5.6(2.8) No chemotherapy: 6.1(2.8)	Surgery Radiation	I-IV	EPIC	EORTC QLQ-C30 CIPN20	Chemotherapy: 93% No Chemotherapy: 89%
Rodriguez 2015 [36] USA	Cross-sectional, population-based	593	Mean 73.8	Only ≥5 years Mean 6.2	Number of treatments	I-III	GLTEQ	PROMIS EQ-5D	–
Thraen-Borowski 2013 [37] USA	Cross-sectional, population-based	832	Mean(SD) 81.5(5.8)	Only ≥5 years Mean(SD) 8.2(1.7)	–	–	CHAMPS	SF-36	52%
Van Roekel 2015 [32]^d^ The Netherlands	Cross-sectional, mono-centric	151	Mean(SD) 69.8(8.7)	2–10 years Mean(SD) 5.7(1.8)	Surgery Radiation Chemotherapy	I-III	SQUASH	EORTC QLQ-C30 WHODAS II CIS HADS	71%
Van Roekel 2016 [33]^d^ The Netherlands	Cross-sectional, mono-centric	145	Mean(SD) 70.0(8.7)	2–10 years Mean(SD) 5.7(1.9)	Surgery Radiation Chemotherapy	I-III	MMOXX1	EORTC QLQ-C30 WHODAS II CIS HADS	–

*Ref*. Reference, *PA* physical activity, *QOL* quality of life, *ACS PA guideline* American Cancer Society physical activity recommendations of at least 150 min of MVPA per week, *SF-36* The Short Form Health Survey, *BMT* Bone marrow transplantation, *GLTEQ* Godin Leisure-Time Exercise Questionnaire, *HW* healty weight, *OW* over weight, *OB* obese, *AAS* The Active Australian Survey, *FACT-C* Functional Assessment of Cancer Therapy-General (FACT-G) plus CRC-specific measurements, *SWLS* Satisfaction With Life Scale, *EPIC* European Prospective Investigation into Cancer Physical Activity Questionnaire, *EORTC QLQ-C30* European Organization for Research and Treatment of Cancer QLQ-C30 questionnaire, *CIPN20* European Organization for Research and Treatment of Cancer QLQ-CIPN20 Chemotherapy-induced peripheral neuropathy questionnaire *PROMIS* Patient-Reported Outcomes Measurement Information System, *EQ-5D* EuroQol Five-Dimension Questionnaire, *CHAMPS* The Community Healthy Activities Model Program for Seniors, *SQUASH* The Short Questionnaire to Assess Health-enhancing physical activity, *WHODAS* World Health Organization Disability Assessment Schedule, *CIS* Checklist Individual Strength, *HADS* Hospital Anxiety and Depression Scale, *MMOXX1* Triaxial MOX activity monitor, ^a^Time since diagnosis at time point of QOL assessment; ^b^Articles based on same study population: American Cancer Society’s Study of Cancer Survivors-II (SCS-II); ^c^Articles based on same study population: All patients diagnosed between 2000 and 2009 and registered in the Patient Reported Outcomes Following Initial treatment and Long term Evaluation of Survivorship (PROFILES registry); ^d^Articles based on same study population: Energy for life after ColoRectal cancer (EnCoRe)

The majority of the studies [9, 30, 32, 33, 35] provided information regarding treatment, such as proportions of patients undergoing surgery, chemotherapy and radiation. Three studies included patients with metastases [9, 30, 31, 34, 35], three studies [32, 33, 36, 38] excluded patients with metastases and one study [37] did not report cancer stage. Four studies [35–38] solely included survivors with a primary diagnosis of CRC, the other studies did not give information about inclusion of survivors with other cancer diagnoses. Four articles [31–33, 36] reported the inclusion of patients with cancer recurrence.

Regarding cancer site, all studies included patients with colon as well as rectal cancers. Five studies [9, 32–34, 36–38] included solely patients with CRC, whilst two studies [30, 31, 35] also included patients with other cancer types. However, the results regarding the association between PA and QOL as well as all figures shown in Table 3 are CRC-specific, only response rates are reported for all cancer types together [30, 31, 35].

### Study design

All included studies were observational in design. Recruitment methods varied across studies, six [9, 30, 31, 34–38] used population-based recruitment, and one [32, 33] was completed in a single institution. Two of the articles [34, 38] were prospective, longitudinal designs assessing PA and/or QOL at multiple points in time, while the remaining eight were cross-sectional [9, 30–33, 35–37].

### Response rate and follow-up rate

The response rates in the aforementioned cross-sectional studies ranged from 33% [30] (not CRC-specific) to 83% [9]. Husson et al. [34] reported a participation of 73% at baseline, 83% for the first and 82% for the second follow-up. In the study of Chambers et al. [38] 56% of the survivors participated in the follow-up, however no information was given regarding baseline participation.

### Assessment and categorization of PA

Apart from one article [34] which measured PA prospectively at three points in time, all other studies [9, 30–33, 35–38] assessed PA only once. One study [33] measured PA by using the Triaxial MOX activity monitor (MMOXX1). The MMOXX1 is able to objectively measure sedentary, standing and PA time. Apart from Blanchard et al. [35] who only reported the adherence or non-adherence to the American Cancer Society (ACS) PA recommendations [39], all other studies [9, 30–32, 34–37] used validated PA instruments relying on self-report. The questionnaire most frequently applied was the Godin Leisure-Time Exercise Questionnaire (GLTEQ) [40]. Several studies [9, 30, 31, 34, 35, 37] used the PA guideline of the ACS [39] to differentiate between active and non-active survivors. The ACS recommends at least 150 min of moderate intensity exercise each week or 75 min of vigorous intensity activity each week or an equivalent combination of both [39]. To further quantify the intensity of the PA, metabolic equivalent hours per week (MET–h/wk) [41] were used in five articles [9, 32–34, 37]. In four of these articles light PA (LPA) was defined as <3 MET–h/wk, whereas moderate to vigorous PA (MVPA) was defined as an intensity of ≥3 MET–h/wk [9, 32, 34, 37]. One article [33] defined PA as >1.5 MET–h/day and did not further differentiate between LPA and MVPA.

### QOL assessment

Quality of life was assessed only at one point in time in most of the studies [9, 30–33, 35–37]. Only the two longitudinal studies [34, 38] assessed QOL at different intervals. Chambers et al. [38] assessed QOL five months post-diagnosis and 5 years after diagnosis. Husson et al. [34] assessed QOL in yearly intervals over a three year period, starting with a baseline average time since diagnosis of 5.1 years. The QOL questionnaires most commonly used were the EORTC QLQ-C30 [9, 32–34] and the SF-36 [30, 31, 35, 37]. Information was collected by mail in six studies [9, 30–32, 34–37], by telephone in five studies [30, 31, 35–38], and in person in one study [33]. One study [32, 33] assessed only some of the EORTC QLQ-C30 subscales and additionally used the Hospital Anxiety and Depression Scale (HADS), the Checklist Individual Strength (CIS), and the World Health Organization Disability Assessment Schedule (WHODAS) questionnaire to assess QOL in CRC survivors.

### Analysis, statistical methods, and clinical relevance

All studies compared CRC survivors who were active with those who were less active or not active. Most of the studies compared survivors who met the ACS PA recommendations to those survivors who did not [9, 30, 31, 34, 35, 37]. Two studies compared different amounts of activity to a non-active reference group of CRC survivors [36, 38]. Some studies compared survivors´ QOL according to higher and lower levels of LPA [32, 37] and/or MVPA [32, 34, 36, 37]. One study additionally compared lower with higher amounts of non-exercise (e.g. gardening) and planned exercise (PA that is planned, structured and repetitive e.g. jogging) [37].

All studies examined possible confounding factors including age, sex, and comorbidities by some sort of multivariable regression modeling or analysis of (co)variance. Six studies adjusted for body mass index (BMI) and only three for smoking. Three studies performed stratified analyses by age, sex, comorbidities, treatment, and BMI for the association between PA and QOL. Two studies [9, 34] reported clinical relevance for the EORTC QLQ-C30. One study reported an overall clinical relevance for the SF-36 of 5–10 score points mean difference [37]. For some studies [30, 31, 35, 38] the clinical relevance was not reported and could not be derived from the available information. Moreover two studies used standard deviations to determine clinical relevance [9, 33].

### Study findings regarding the association between PA and QOL

According to the included studies, 35–80% of the CRC survivors met the ACS PA recommendations (Table 3). Tables 4 and 5 and the Additional file 2: Table S2 and Additional file 3: Table S3 show the study specific results regarding the association between PA and QOL according to type of analysis and type of QOL instrument. Since the included studies used various QOL questionnaires, which differ in included scales, not all studies contributed to the results on every outcome and are thus not considered when summarizing the respective findings.

**Table 4:** Association of PA and QOL - Active vs. non-active

		Statistical significance (*p* <0.05) and clinical relevance										
		+/−: significant positive/negative association ns: not statistically significant .: not reported						^a,b,c^clinical relevance				
Study	C30	QL		PF		RF		EF		SF		CF
Husson 2015 [34]	Meeting vs. not meeting ACS PA guideline, Interindividual^d^	+^b^		+^b^		+^b^		+^b^		+^b^		+
	Meeting vs. not meeting ACS PA guideline, Intraindividual^e^	+		+		+		ns		ns		ns
Mols 2015 [9]	Meeting vs. not meeting ACS PA guideline	+^c^		+^c^		+^c^		+^c^		+^c^		+^c^
Study	SF-36	PF	RP	BP	SF	MH	RE	VT	GH	GCS	PCS	MCS
Blanchard 2004 [35]	Meeting vs. not meeting ACS PA guideline	.	.	.	.	.	.	.	.	+^c^	.	.
Blanchard 2008 [30]	Meeting vs. not meeting ACS PA guideline	.	.	.	.	.	.	.	.	+^c^	.	.
Thraen-Borowski 2013 [37]	Meeting vs. not meeting ACS PA guideline	+^a^	+^a^	+	+^a^	ns	ns	+^a^	+^a^	.	.	.
Study	FACT-C/ SWLS	PWB		SWB		EWB		FWB		CCS		SWLS
Chambers 2012 [38]	Sedentary - Ref. Insufficiently active (1–149 min/wk)	ns		ns		ns		ns		ns		ns
	Sufficiently active (≥150 min/wk)	ns		ns		ns		ns		ns		ns
Study	PROMIS/ EQ-5D	Physical HRQOL				Mental HRQOL				Overall HRQOL		
Rodriguez 2015 [36]	PA min/wk No PA - Ref. ≤60, 61–149, 150–249, 250+	+^c^ (≤60, 61–149, 150–249)				ns				+^c^ (≤60, 61–149, 150–249)		

*PA* physical activity, *QOL* quality of life, *C30* (European Organization for Research and Treatment of Cancer QLQ-C30 questionnaire) *QL* global quality of life, *PF* physical functioning, *RF* role functioning, *EF* emotional functioning, *SF* social functioning, *CF* cognitive functioning, *ACS PA guideline* American Cancer Society physical activity recommendations of at least 150 min of MVPA per week, *SF-36* (The Short Form Health Survey) *PF* physical functioning, *RP* role limitations due to physical health problems, *BP* bodily pain, *SF* social functioning, *MH* general mental health, *RE* role limitations due to emotional problems, *VT* vitality, *GH* general health perceptions, *GCS* global health composite score, *PCS* physical composite score, *MCS* mental composite score, *FACT-C* (Functional Assessment of Cancer Therapy - Colorectal Cancer) *PWB* physical well-being, *SWB* social well-being, *EWB* emotional well-being, *FWB* functional well-being, *CCS* colorectal cancer scale, *SWLS* (Satisfaction with Life Scale), *Ref*. Reference, *min/wk* minutes per week, *PROMIS* (Patient-Reported Outcomes Measurement Information System), *EQ-5D* (EuroQol Five-Dimension Questionnaire), ^a^clinical relevance reported by authors; ^b^clinical relevance calculated by RE; ^c^clinical relevance: no values, no cut-off for calculation available; ^d^interindividual: patients average amount of PA/ average level PA of total group; ^e^intraindividual: patients PA level at one time point/ patients average PA level

**Table 5:** Association of PA and QOL - Different levels of PA and linear association

		Statistical significance (p <0.05) and clinical relevance										
	Different levels of PA	+/−: significant positive/negative association ns: not statistically significant .: not reported						^a,b,c^clinical relevance				
Study	C30	QL		PF		RF		EF		SF		CF
Van Roekel 2015 [32]	>LPA (Q4 = ≥23.0 h/wk) vs. <LPA (Q1 = ≤2.0 h/wk)	ns		+^b^		+^b^		.		ns		.
	>LPA (Q3 = 10.0-22.0 h/wk) vs. <LPA (Q1 = ≤2.0 h/wk)	.		.		.		.		.		.
	>MVPA (Q4 = ≥15.5 h/wk) vs. <MVPA (Q1 = ≤4.3 h/wk)	ns		+^b^		ns		.		ns		.
	>MVPA (Q3 = 8.7-15.0 h/wk) vs. <MVPA (Q1 = ≤4.3 h/wk)	ns		.		+^b^		.		+^b^		.
Study	SF-36	PF	RP	BP	SF	MH	RE	VT	GH	GCS	PCS	MCS
Thraen-Borowski 2013 [37]	>MVPA (Q4 = ≥11.3 h/wk) vs. <MVPA (Q1 = 0.0 h/wk)	.	.	.	.	.	.	.	.	.	+^b^	ns
	>LPA (Q4 = ≥13.0 h/wk) vs. <LPA (Q1 = ≤1.5 h/wk)^d^	.	.	.	.	.	.	.	.	.	ns	ns
	>LPA (Q4 = ≥9.0 h/wk) vs. <LPA (Q1 = 0.0 h/wk)^e^	.	.	.	.	.	.	.	.	.	+^b^	+^b^
	>Planned exercise^f^ (Q4 = ≥9.5 h/wk) vs. <Planned exercise (Q1 = 0.0 h/wk)	.	.	.	.	.	.	.	.	.	+^b^	ns
	>Non-exercise^g^ (Q4 = ≥16.5 h/wk) vs. <Non-exercise (Q1 = ≤1.6 h/wk)	.	.	.	.	.	.	.	.	.	+	ns
Study	WHODAS/ CIS/ HADS	DIS				FA				DIST		
Van Roekel 2015 [32]	>LPA (Q4 = ≥23.0 h/wk) vs. <LPA (Q1 = ≤2.0 h/wk)	–^c^				ns				ns		
	>LPA (Q3 = 10.0-22.0 h/wk) vs. <LPA (Q1 = ≤2.0 h/wk)	ns				–^c^				ns		
	>MVPA (Q4 = ≥15.5 h/wk) vs. <MVPA (Q1 = ≤4.3 h/wk)	ns				ns				ns		
	>MVPA (Q3 = 8.7-15.0 h/wk) vs. <MVPA (Q1 = ≤4.3 h/wk)	–^c^				–^c^				–^c^		
Study	PROMIS/ EQ-5D	Physical HRQOL				Mental HRQOL				Overall HRQOL		
Rodriguez 2015 [36]	MVPA min/wk. No MVPA - Ref. ≤60, 61–149, 150+	ns				ns				+^c^ (61–149, 150+) ns (≤ 60)		
Linear association PA and QOL (continuous results)												
Study	C30	QL		PF		RF		EF		SF		CF
Hussonn 2015 [34]	Continuous: Additional hour of MVPA/wk., Interindividual^h^	+		+		+		+		+		+
	Continuous: Additional hour of MVPA/wk., Intraindividual^i^	ns		+		ns		ns		ns		+
Van Roekel 2016 [33]	Single-variable model, PA^j^	ns		+		ns		.		ns		.
	Partition model, PA^k^	ns		+		ns		.		ns		.
	Substituting 1 h/day of sedentary time with PA	ns		+^a^		ns		.		ns		.
	Substituting 1 h/day of standing time with PA	ns		ns		ns		.		ns		.
Study	WHODAS/ CIS/ HADS	DIS				FA		ANX				DEP
Van Roekel 2016 [33]	Single-variable model PA^j^	–^c^				ns		ns				ns
	Partition model PA^k^	ns				ns		ns				ns
	Substituting 1 h/day of sedentary time with PA	ns				ns		ns				ns
	Substituting 1 h/day of standing time with PA	ns				ns		ns				ns

*PA* physical activity, *QOL* quality of life, C30 (European Organization for Research and Treatment of Cancer QLQ-C30 questionnaire), *QL* global quality of life, *PF* physical functioning, *RF* role functioning, *EF* emotional functioning, *SF* social functioning, *CF* cognitive functioning; > more; < less, *LPA* light physical activity (<3 MET), *Q* Quartile, *h/wk* hours per week, *MVPA* moderate to vigorous physical activity (≥3 MET), *SF-36* (The Short Form Health Survey) *PF* physical functioning, *RP* role limitations due to physical health problems, *BP* bodily pain, *SF* social functioning, *MH* general mental health, *RE* role limitations due to emotional problems, *VT* vitality, *GH* general health perceptions, *GCS* global health composite score, *PCS* physical composite score, *MCS* mental composite score, *WHODAS* (World Health Organization Disability Assessment Schedule II) *DIS* disability, *CIS* (Checklist Individual Strength) *FA* fatigue, *HADS* (Hospital Anxiety and Depression Scale), *DIST* distress, *ANX* anxiety, *DEP* depression, *PROMIS* (Patient-Reported Outcomes Measurement Information System), *EQ-5D* (EuroQol Five-Dimensions Questionnaire), *Ref*. Reference, ^a^clinical relevance reported by authors; ^b^clinical relevance calculated by RE; ^c^clinical relevance: no values, no cut-off for calculation available; ^d^participants reported LPA and MVPA; ^e^participants reported only LPA; ^f^intentional exercise e.g. jogging; ^g^non-intentional exercise e.g. gardening; ^h^interindividual: patients average amount of PA/ average level PA of total group; ^i^intraindividual: patients PA level at one time point/ patients average PA level; ^j^PA was entered separately in a single confounder-adjusted model, without adjustment for any of the other activities (sedentary, standing); ^k^all activity categories (sedentary, standing, PA) were entered simultaneously in a single confounder-adjusted model, to estimate independent associations of each activity category

### Physically active vs. not active

Five of the six studies which compared active with non-active CRC survivors, found positive associations between PA and QOL (Table 4). Regarding specific subscales, homogenous results were found for global QOL, which was positively associated with PA in all of the five studies which investigated global QOL. Differences in global QOL between physically active versus non-active survivors were clinically relevant in two [34, 37] of the five studies. Three out of four studies reported a positive association between PA and physical functioning, of these two [34, 37] associations were of clinical relevance. Two studies [30, 35] did not report any results on physical functioning. In contrast, results for role and social functioning were more heterogeneous and less often statistically significant.

### Different levels of PA and linear association of PA and QOL

Table 5 shows the results from studies examining the association between multiple levels of PA and QOL. Higher QOL was associated with both, lower and higher levels of PA intensity but the association between PA and QOL depended on the specific QOL dimension. For instance, survivors who had higher levels of LPA reported significantly and clinically relevant higher physical functioning than survivors who had lower LPA levels [32, 37], but no association was found between global QOL, social functioning, and LPA [32], respectively. Positive associations between MVPA and physical functioning were found in two [32, 37] of three [32, 36, 37] studies. Survivors who reported higher MVPA levels reported significantly and clinically relevant higher physical functioning compared to survivors who had lower MVPA levels [32, 37].

When assessing PA as a continuous variable, significant positive associations of MVPA with higher global QOL, physical, emotional, social, and cognitive functioning were found [34]. Van Roekel et al. reported significant positive associations between PA time (hour/day) and physical functioning and disability, however, no associations were found for global QOL, role and social functioning, fatigue, anxiety, and depression [33].

### Further subgroup analyses and changes in the association of PA and QOL over time

Only the study by van Roekel et al. provided results stratified for age [33] and sex [32, 33] (Additional file 2: Table S2). The association between PA and QOL did not differ between younger and older survivors. However, the association between LPA/PA and QOL seemed to be stronger among women than among men. Women who had higher LPA levels reported significantly and clinically relevant higher physical, role, and social functioning and significantly less disability compared to women who had lower LPA levels. The association of PA with global QOL, fatigue, and distress was not statistically significant. When substituting one hour of sedentary time with PA, PA was clinically and significantly associated with higher physical functioning and lower disability in women. However, PA was not associated with global QOL, role and social functioning, fatigue, anxiety, and depression when substituting one hour of sedentary time with PA. In both investigations no significant associations were found in men.

Van Roekel et al. [32, 33] reported heterogeneous results for the association between LPA/PA and QOL stratified by number of comorbidities. Survivors with ≥2 comorbidities who reported higher levels of LPA reported significantly and clinically relevant higher physical and role functioning and significantly less disability than survivors with lower levels of LPA. No associations were observed between higher levels of LPA and global QOL, social functioning, fatigue, and distress. No associations were reported for LPA levels and any QOL scales for survivors with <2 comorbidities [32]. In contrast, when using sedentary time or standing time as a proxy measures of (lack of) PA, none of the QOL scales were associated with PA in neither survivors with <2 nor survivors ≥2 comorbidities [33].

Heterogeneous results were also reported regarding the association between PA and QOL with respect to BMI. According to van Roekel et al. [33] non-obese survivors who were physically active reported higher global QOL, lower depression and anxiety than less active non-obese survivors. No association between PA and QOL was found among obese survivors. In contrast, in the study of Blanchard et al. [31] no associations between PA and QOL were found according to BMI.

Survivors without chemotherapy treatment who were physically active scored significantly lower on the sensory, motor, and autonomic scale of the European Organization for Research and Treatment of Cancer QLQ-CIPN20 Chemotherapy-induced peripheral neuropathy questionnaire (CIPN 20), compared to non-active survivors [9]. The association between PA and QOL among CRC survivors with chemotherapy treatment did not substantially differ, only no significant associations were found for PA and the autonomic scale. In both, survivors with and without chemotherapy treatment, associations between PA and the motor scale were of clinical relevance.

Only one study assessed PA and QOL at various points in time among the same patients [34]. In CRC survivors who were physically active over a three years period, role and social functioning improved whereas role and social functioning declined in non-active survivors. No associations were found between persistent PA and global QOL, physical, emotional, and cognitive functioning [34].

## Discussion

### Key findings

The results from this systematic review demonstrate that long-term CRC survivors who were more physically active generally reported higher QOL than non-active survivors. Moreover, different PA levels such as LPA and MVPA seemed to be associated with QOL in long-term CRC survivors. The association between PA and QOL associations seemed to be stronger among women than among men. However, no general conclusion can be drawn, since only few studies performed specific subgroup analyses.

To our knowledge, three review articles [10, 42, 43] have been published on the associations between PA and QOL in CRC survivors. However, the articles [10, 42, 43] published so far were based on studies which mainly included short-term CRC survivors and no systematic review has specifically focused on long-term CRC survivors. The results which were found in this review article are quite homogenous. Eight of the ten included articles found associations between PA and QOL, whereas the results of the previous reviews are more inconsistent. In line with our findings, Lynch et al. [10] who included short-term and long-term survivors, also reported associations between PA and QOL in observational studies. Otto et al. [43] reported that the association between PA and QOL was stable over time but only focused on short-term survivors. In contrast, the review article and meta-analysis of Cramer et al. [42] which included only short-term survivors did not find an association between PA and HRQOL. The inconsistent findings between our review and the previous review articles might be explained in parts by the different study population characteristics. The most obvious difference is the varying time since diagnosis. Due to the heterogeneous findings, it remains unclear whether the overall effect of PA on QOL differs for short-term and long-term CRC patients.

### Limitations

Even though the majority of the studies, included in this review had large sample sizes, were population based, examined possible confounding factors like age, sex, and comorbidities and used validated QOL and PA questionnaires, most of the included studies have some shortcomings which might limit their contributions to existing evidence.

Nine of ten included articles assessed the association between PA and QOL using a cross-sectional design. For these studies we cannot assume causality, only an association between PA and QOL at one point in time. Moreover, only few studies reported results stratified by important covariates such as age, sex, or treatment. Although the focus of this review article was on long-term CRC survivors, only two studies [36, 37] were identified that solely included long-term CRC survivors. All other studies included short and long-term survivors with a mean of 5 or more years since diagnosis. Thus, results of the review are in parts not only based on long-term survivors. Since we did not include CRC survivors irrespective of time since diagnosis, but rather focused on long-term survivors, testing for a moderating time since treatment was not possible.

Given the older age of long-term CRC survivors and the higher number of comorbid chronic conditions, it may be reasonable to assume that the magnitude of the effect of PA on QOL would be smaller, relative to short-term survivors. However, in the majority of the included studies this effect appears to remain statistically significant.

A further limitation of the current studies is that the majority used self-reported PA measures. Only one study [33] used an activity monitor to assess PA. In this context, information bias such as reporting bias might occur in studies relying on self-reported PA levels or by only assessing leisure time PA, but not work-related PA. Furthermore, there were differences in the measurement tools used to assess QOL which may also introduce some information bias. Some studies [9, 32–34, 38] used cancer-specific QOL questionnaires and other studies [30, 31, 35–37] used general QOL instruments. Therefore the differences in the QOL assessment might limit the comparability of the results. In addition, many QOL instruments specifically designed for cancer patients under active treatment, such as the EORTC QLQ-C30 and the FACT-G, with their supplementary condition-specific or symptom-specific modules, are not entirely appropriate or sufficient for assessing the experience of disease free cancer survivors.

Furthermore, the sensitivity of QOL instruments and scales to detect subtle differences in QOL may have had an impact on the results. For example, two of the included articles [31, 38] did not find any association between PA and QOL. An explanation for the non-significant results in the article of Chambers et al. [38] might be the use of specific questionnaires (FACT-C, SWLS), which might not be sufficiently sensitive. The other article [31] not finding significant associations is based on the same study population as another included article [30] which found associations between PA and QOL. However, the article of Blanchard et al. which did not report significant results [31], did not present the results for the general associations of PA with QOL again, but only reported the association between PA and QOL stratified by BMI. Therefore BMI might have been a confounding factor.

Due to the heterogeneity of the study methods and results, no meta-analysis could be performed.

As a result of early detection and treatment, more and more CRC patients are becoming long-term survivors [11]. Therefore, there is a need to maintain or improve the QOL of these patients. Previous studies suggest that counselling CRC survivors to engage in regular PA is warranted to improve the prognosis of those patients. The results of this review further support a positive association between PA and QOL, however most included studies have some limitations regarding the study design, thus results should be interpreted with caution.

To overcome the aforementioned limitations and to provide more evidence regarding the causality of a potential beneficial effect of PA on QOL, there is an urgent need for more prospective studies assessing PA and QOL at multiple points in time, preferably by using a randomized controlled trial design (e.g. [44]). Also future studies should more often incorporate a prospective and validated assessment of PA, for example by including objective activity monitoring, in order to learn more about the dose-response relationship of PA and QOL. More attention should be given to potential effect modification by age, gender, type of treatment, stage, and other clinically relevant patients´ characteristics. As health-related QOL represents a multi-dimensional concept, studies should use validated and reliable QOL instruments for which clinically important differences have been established and which cover both cancer-specific and general QOL measures regarding psychological as well as physical aspects. In order to differentiate potential specific effects of PA on QOL in CRC survivors from general effects of PA, additional studies comparing CRC survivors with an age-matched sample from the general population as controls might be warranted.

Future studies including the aforementioned suggestions may help to identify survivors who will benefit most from PA intervention and to identify the point in time and the level of PA that may be beneficial to CRC survivors. Therefore, we may potentially be able to provide more specific and adequate recommendations regarding PA in CRC patients.

## Conclusions

Despite the limitations of the existing evidence, the results of our systematic review indicate that overall, PA is associated with better QOL in CRC survivors. Moreover, different PA levels such as LPA and MVPA seemed to be associated with QOL in long-term CRC survivors, therefore it might be beneficial for long-term CRC survivors to be physically active. Further prospective studies and randomized controlled trials are needed to further evaluate and confirm the causality of the association between PA and QOL specifically in long-term CRC survivors, in order to provide more solid evidence for individual PA recommendations.

## Additional files

Additional file 1: **Table S1.** Search terms. (DOCX 13 kb)

Additional file 2: **Table S2.** Association of PA and QOL – Subgroup analyses. (DOCX 43 kb)

Additional file 3: **Table S3.** Association of PA and QOL - Symptom scales. (DOCX 18 kb)

## Abbreviations

ACS: American Cancer Society
BMI: Body mass index
CIPN20: European Organization for Research and Treatment of Cancer QLQ-CIPN20 Chemotherapy-induced peripheral neuropathy questionnaire
CIS: Checklist Individual Strength
CRC: Colorectal cancer
EORTC QLQ-C30: European Organization for Research and Treatment of Cancer QLQ-C30 questionnaire
EQ-5D: EuroQol Five-Dimension Questionnaire
FACT-C: Functional Assessment of Cancer Therapy - Colorectal Cancer
GLTEQ: Godin Leisure-Time Exercise Questionnaire
HADS: Hospital Anxiety and Depression Scale
HRQOL: Overall health related quality of life
LPA: Light physical activity
MET–h/wk: Metabolic equivalent hours per week
MMOXX1: Triaxial MOX activity monitor
MVPA: Moderate to vigorous physical activity
PA: Physical activity
PROMIS: Patient-Reported Outcomes Measurement Information System
QOL: Quality of life
SF-36: The Short Form Health Survey
SWLS: Satisfaction With Life Scale
WHODAS: World Health Organization Disability Assessment Schedule
