# Supplementary material for: Quality of life and physical activity in long-term (≥5 years post-diagnosis) colorectal cancer survivors - systematic review
Source: Health Qual Life Outcomes. 2018 Jun 1;16:112. doi: 10.1186/s12955-018-0934-7 (PMC5984808; doi:10.1186/s12955-018-0934-7)
Supplement: Supplementary file 1 — Table S1. Search terms. (DOCX 13 kb) [file 12955_2018_934_MOESM1_ESM.docx]

**Additional file 1: Table S1: Search terms**

The following combinations of search terms were used:

(colorectal cancer OR colorectal neoplasms OR colorectal carcinoma OR colon cancer OR colon carcinoma OR rectal cancer OR rectal carcinoma OR rectal neoplasms OR colonic neoplasms OR intestinal cancer OR intestinal neoplasms OR lower gastrointestinal tract OR bowel cancer)

AND

(quality of life OR well-being OR mental health OR QOL OR HRQOL OR life quality OR qualities of life OR life satisfaction OR personal satisfaction)

AND

(motor activity OR physical activity OR exercise OR sedentary lifestyle).

In the database PubMed, the following Mesh terms were used additionally:

(colorectal neoplasms OR intestinal neoplasms OR colon OR rectum OR rectal neoplasms OR colonic neoplasms OR lower gastrointestinal tract)

AND

(quality of life OR mental health OR personal satisfaction)

AND

(motor activity OR exercise OR sedentary lifestyle).
