# Supplementary material for: Quality of life and physical activity in long-term (≥5 years post-diagnosis) colorectal cancer survivors - systematic review
Source: Health Qual Life Outcomes. 2018 Jun 1;16:112. doi: 10.1186/s12955-018-0934-7 (PMC5984808; doi:10.1186/s12955-018-0934-7)
Supplement: Supplementary file 2 — Table S2. Association of PA and QOL – Subgroup analyses. (DOCX 43 kb) [file 12955_2018_934_MOESM2_ESM.docx]

**Additional file 2: Table S2: Association of PA and QOL – Subgroup analyses**

|  |  | **Statistical significance (p<0.05) and clinical relevance** | | | | | | | | | | | | | | | | | |
| --- | --- | --- | --- | --- | --- | --- | --- | --- | --- | --- | --- | --- | --- | --- | --- | --- | --- | --- | --- |
|  | **Subgroup analyses** | +/– significant positive/negative association  ns: not statistically significant  .: not reported | | | | | | | | | ^a,b,c^clinical relevance | | | | | | | | |
| **Study** | **C30** | **QL** | | **PF** | | | | **RF** | | | **EF** | | | **SF** | | | | **CF** | |
|  | ***Sex*** |  | |  | | | |  | | |  | | |  | | | |  | |
| Van Roekel  2015[32] | >LPA (Q4= ≥23.0h/wk) vs. <LPA (Q1= ≤2.0h/wk)  Women | ns | | +^b^ | | | | +^b^ | | | . | | | +^b^ | | | | . | |
|  | Men | ns | | ns | | | | ns | | | . | | | ns | | | | . | |
|  | ***Comorbidities*** |  | |  | | | |  | | |  | | |  | | | |  | |
|  | >LPA (Q4= ≥23.0h/wk) vs. <LPA (Q1= ≤2.0h/wk)  ≥2 Comorbidities | ns | | +^b^ | | | | +^b^ | | | . | | | ns | | | | . | |
|  | <2 Comorbidities | ns | | ns | | | | ns | | | . | | | ns | | | | . | |
|  | ***Sex*** |  | |  | | | |  | | |  | | |  | | | |  | |
| Van Roekel  2016[33] | Substituting 1 h/day of sedentary time with PA  Women | ns | | +^a^ | | | | ns | | | . | | | ns | | | | . | |
|  | Men | ns | | ns | | | | ns | | | . | | | ns | | | | . | |
|  | Substituting 1 h/day of standing time with PA  Women | ns | | ns | | | | ns | | | . | | | ns | | | | . | |
|  | Men | ns | | ns | | | | ns | | | . | | | ns | | | | . | |
|  | ***Age*** |  | |  | | | |  | | |  | | |  | | | |  | |
|  | Substituting 1 h/day of sedentary time with PA  <70 years | ns | | ns | | | | ns | | | . | | | ns | | | | . | |
|  | ≥70 years | ns | | ns | | | | ns | | | . | | | ns | | | | . | |
|  | Substituting 1 h/day of standing time with PA  <70 years | ns | | ns | | | | ns | | | . | | | ns | | | | . | |
|  | ≥70 years | ns | | ns | | | | ns | | | . | | | ns | | | | . | |
|  | ***BMI*** |  | |  | | | |  | | |  | | |  | | | |  | |
|  | Substituting 1 h/day of sedentary time with PA  Non-obese | +^a^ | | ns | | | | ns | | | . | | | ns | | | | . | |
|  | Obese | ns | | ns | | | | ns | | | . | | | ns | | | | . | |
|  | Substituting 1 h/day of standing time with PA  Non-obese | ns | | ns | | | | ns | | | . | | | ns | | | | . | |
|  | Obese | ns | | ns | | | | ns | | | . | | | ns | | | | . | |
|  | ***Comorbidities*** |  | |  | | | |  | | |  | | |  | | | |  | |
|  | Substituting 1 h/day of sedentary time with PA  <2 comorbidities | ns | | ns | | | | ns | | | . | | | ns | | | | . | |
|  | ≥2 comorbidities | ns | | ns | | | | ns | | | . | | | ns | | | | . | |
|  | Substituting 1 h/day of standing time with PA  <2 comorbidities | ns | | ns | | | | ns | | | . | | | ns | | | | . | |
|  | ≥2 comorbidities | ns | | ns | | | | ns | | | . | | | ns | | | | . | |
|  |  |  | |  | | | |  | | |  | | |  | | | |  | |
| **Table S2: Association of PA and QOL – Subgroup analyses** *(continued)* | | | | | | | | | | | | | | | | | | | |
| **Study** | **CIPN20** | **Sensory** | | | | | | **Motor** | | | | | | **Autonomic** | | | | | |
|  | ***Treatment*** |  | | | | | |  | | | | | |  | | | | | |
| Mols  2015[9] | Meeting vs. not meeting ACS PA guideline  CT, PA vs. CT, no PA | + | | | | | | + | | | | | | ns | | | | | |
|  | Meeting vs. not meeting ACS PA guideline  No CT, PA vs. no CT, no PA | +^a^ | | | | | | + | | | | | | + | | | | | |
| **Study** | **SF-36** | **PF** | **RP** | | **BP** | | **SF** | | **MH** | **RE** | | **VT** | **GH** | | **GCS** | | **PCS** | | **MCS** |
|  | ***BMI*** |  |  | |  | |  | |  |  | |  |  | |  | |  | |  |
| Blanchard  2010[31] | Meeting vs. not meeting ACS PA guideline  Healthy weight | . | . | | . | | . | | . | . | | . | . | | . | | ns | | ns |
|  | Overweight | . | . | | . | | . | | . | . | | . | . | | . | | ns | | ns |
| **Study** | **WHODAS/ CIS/ HADS** | **DIS** | | | | | | **FA** | | | | | | **DIST** | | | | | |
|  | ***Sex*** |  | | | | | |  | | | | | |  | | | | | |
| Van Roekel 2015[32] | >LPA (Q4= ≥23.0h/wk) vs. <LPA (Q1= ≤2.0h/wk)  Women | –^c^ | | | | | | ns | | | | | | ns | | | | | |
|  | Men | ns | | | | | | ns | | | | | | ns | | | | | |
|  | ***Comorbidities*** |  | | | | | |  | | | | | |  | | | | | |
|  | >LPA (Q4= ≥23.0h/wk) vs. <LPA (Q1= ≤2.0h/wk)  ≥2 comorbidities | –^c^ | | | | | | ns | | | | | | ns | | | | | |
|  | <2 comorbidities | ns | | | | | | ns | | | | | | ns | | | | | |
| **Study** | **WHODAS/ CIS/ HADS** | **DIS** | | | | **FA** | | | | | **ANX** | | | | | **DEP** | | | |
|  | ***Sex*** |  | | | |  | | | | |  | | | | |  | | | |
| Van Roekel 2016[33] | Substituting 1 h/day of sedentary time with PA  Women | –^a^ | | | | ns | | | | | ns | | | | | ns | | | |
|  | Men | ns | | | | ns | | | | | ns | | | | | ns | | | |
|  | Substituting 1 h/day of standing time with PA  Women | ns | | | | ns | | | | | ns | | | | | ns | | | |
|  | Men | ns | | | | ns | | | | | ns | | | | | ns | | | |
|  | ***Age*** |  | | | |  | | | | |  | | | | |  | | | |
|  | Substituting 1 h/day of sedentary time with PA  <70 years | ns | | | | ns | | | | | ns | | | | | ns | | | |
|  | ≥70 years | ns | | | | ns | | | | | ns | | | | | ns | | | |
|  | Substituting 1 h/day of standing time with PA  >70 years | ns | | | | ns | | | | | ns | | | | | ns | | | |
|  | ≥70 years | ns | | | | ns | | | | | ns | | | | | ns | | | |
|  | ***BMI*** |  | | | |  | | | | |  | | | | |  | | | |
|  | Substituting 1 h/day of sedentary time with PA  Non-obese | ns | | | | ns | | | | | – | | | | | – | | | |
|  | Obese | ns | | | | ns | | | | | ns | | | | | ns | | | |
|  | Substituting 1 h/day of standing time with PA  Non-obese | ns | | | | ns | | | | | –^a^ | | | | | – | | | |
|  | Obese | ns | | | | ns | | | | | ns | | | | | ns | | | |
| **Table S2: Association of PA and QOL – Subgroup analyses** *(continued)* | | | | | | | | | | | | | | | | | | | |
|  | ***Comorbidities*** |  | | | |  | | | | |  | | | | |  | | | |
|  | Substituting 1 h/day of sedentary time with PA  <2 comorbidities | ns | | | | ns | | | | | ns | | | | | ns | | | |
|  | ≥2 comorbidities | ns | | | | ns | | | | | ns | | | | | ns | | | |
|  | Substituting 1 h/day of standing time with PA  <2 comorbidities | ns | | | | ns | | | | | ns | | | | | ns | | | |
|  | ≥2 comorbidities | ns | | | | ns | | | | | ns | | | | | ns | | | |

**PA:** physical activity; **QOL:** quality of life; **C30** (European Organization for Research and Treatment of Cancer QLQ-C30 questionnaire) *QL*: global quality of life, *PF*: physical functioning, *RF*: role functioning, *EF*: emotional functioning, *SF*: social functioning, *CF*: cognitive functioning; **>:** more; **<:** less; **LPA:** light physical activity (<3 METs); **Q:** Quartile; **h/wk:** hours per week; **h/day:** hours per day; **BMI:** body mass index; **CIPN20** (European Organization for Research and Treatment of Cancer QLQ-CIPN20 Chemotherapy-induced peripheral neuropathy); **ACS PA guideline:** American Cancer Society physical activity recommendations of at least 150 minutes of MVPA per week; **CT:** Chemotherapy; **SF-36** (THE SHORT-FORM-36 HEALTH SURVEY) *PF*: physical functioning, RP: role limitations due to physical health problems, *BP*: bodily pain, *SF*: social functioning, *MH*: general mental health, *RE*: role limitations due to emotional problems, *VT*: vitality, *GH*: general health perceptions, *GCS*: global health composite score, *PCS*: physical composite score, *MCS*: mental composite score; **WHODAS** (World Health Organization Disability Assessment Schedule II) *DIS*: disability; **CIS** (Checklist Individual Strength) *FA*: fatigue; **HADS** (Hospital Anxiety and Depression Scale) *DIST*: distress, *ANX*: anxiety, *DEP*: depression; **^a^**clinical importance reported by authors; **^b^**calculated by RE; **^c^**no values, no cut-off for calculation available
