# Supplementary material for: Quality of life and physical activity in long-term (≥5 years post-diagnosis) colorectal cancer survivors - systematic review
Source: Health Qual Life Outcomes. 2018 Jun 1;16:112. doi: 10.1186/s12955-018-0934-7 (PMC5984808; doi:10.1186/s12955-018-0934-7)
Supplement: Supplementary file 3 — Table S3. Association of PA and QOL - Symptom scales. (DOCX 18 kb) [file 12955_2018_934_MOESM3_ESM.docx]

| **Additional file 3: Table S3: Association of PA and QOL - Symptom scales** | | | | | | | | | | | | |
| --- | --- | --- | --- | --- | --- | --- | --- | --- | --- | --- | --- | --- |
|  | | **Statistical significance (p<0.05) and clinical relevance** | | | | | | | | | |  |
|  |  | +/–: significant positive/negative association  ns: not statistically significant | | | | | ^a,b,c^clinical relevance | | | | |  |
| **Study** | | **C30** | **Pain** | **Fatigue** | **Nausea,**  **Vomiting** | **Appetite loss** | **Constipation** | | **Diarrhea** | **Dyspnea** | **Insomnia** | **Financial difficulties** |
| Mols  2015[9] | | Meeting vs. not meeting ACS PA guideline  Low neuropathy^d^ | ‒^a^ | ‒ | ns | ‒ | ns | | ‒^a^ | ‒^a^ | ns | ns |
|  | | Meeting vs. not meeting ACS PA guideline  High neuropathy^e^ | ‒^a^ | ‒^a^ | ns | ‒ | ns | | ‒^a^ | ‒^a^ | ns | ns |
|  | | Meeting vs. not meeting ACS PA guideline | ‒ | ‒ | ns | ‒ | ns | | ‒ | ‒ | ns | ns |

**PA:** physical activity; **QOL:** quality of life; **C30**: European Organization for Research and Treatment of Cancer QLQ-C30 questionnaire; **ACS PA guideline:** American Cancer Society physical activity recommendations of at least 150 minutes of MVPA per week; **^a^**clinical importance reported by authors; **^b^**calculated by RE; **^c^**no values, no cut-off for calculation available; **^d^**70% of patients with lowest scores of CIPN; **^e^**30% of patients with highest scores of CIPN
